# Institutional public private partnerships for core health services: evidence from Italy

**DOI:** 10.1186/1472-6963-11-82

**Published:** 2011-04-19

**Authors:** Giulia Cappellaro, Francesco Longo

**Affiliations:** 1 Cambridge Judge Business School, University of Cambridge, Trumpington Street, Cambridge, CB2 1AG, UK; 2 Centre for Research on Health and Social Care Management, Università Commerciale Luigi Bocconi, Via Roentgen 1, 20136 Milan, Italy; 3 Department of Public Management and Institutional Analysis, Università Commerciale Luigi Bocconi, Via Roentgen 1, 20136 Milan, Italy

## Abstract

**Background:**

Public-private partnerships (PPPs) are potential instruments to enable private collaboration in the health sector. Despite theoretical debate, empirical analyses have thus far tended to focus on the contractual or project dimension, overlooking institutional PPPs, i.e., formal legal entities run by proper corporate-governance mechanisms and jointly owned by public and private parties for the provision of public-health goods. This work aims to fill this gap by carrying out a comparative analysis of the reasons for the adoption of institutional PPPs and the governance and managerial features necessary to establish them as appropriate arrangements for public-health services provisions.

**Methods:**

A qualitative analysis is carried out on experiences of institutional PPPs within the Italian National Health Service (*Sistema Sanitario Nazionale*, SSN). The research question is addressed through a contextual and comparative embedded case study design, assuming the entire population of PPPs (4) currently in force in one Italian region as the unit of analysis: (i) a rehabilitation hospital, (ii), an orthopaedic-centre, (iii) a primary care and ambulatory services facility, and (iv) a health- and social-care facility. Internal validity is guaranteed by the triangulation of sources in the data collection phase, which included archival and interview data.

**Results:**

Four governance and managerial issues were found to be critical in determining the positive performance of the case examined: (i) a strategic market orientation to a specialised service area with sufficient potential demand, (ii) the allocation of public capital assets and the consistent financial involvement of the private partner, (iii) the adoption of private administrative procedures in a regulated setting while guaranteeing the respect of public administration principles, and (iv) clear regulation of the workforce to align the contracts with the organisational culture.

**Conclusions:**

Findings suggests that institutional PPPs enable national health services to reap great benefits when introduced as a complement to the traditional public-service provisions for a defined set of services and goals.

## Background

In the past 20 years, the notion of public service provision has undergone a radical revision, ultimately strengthened by a shift towards the view of the state as a regulator and purchaser rather than purely a provider of services. Various provision forms have been adopted: in-house production; outsourcing to other public administrations; contracting out of peripheral, intermediate, or final services to private providers; public-private partnerships (PPPs) and full privatisation [1,2].

PPPs have long been considered as the form of provision able to maximise the gains deriving from private production, while preserving the collective goals of the public realm. However, these hybrid solutions may be rather difficult to design and implement, due to the heterogeneous - and potentially conflicting - missions, goals, organisational cultures and legal frameworks adopted by the partners.

The overarching aim of this paper is to investigate the motivations for the adoption of institutional PPPs and the governance and managerial features that could make them effective. These dimensions are strictly interdependent and reinforce each other. Indeed, the extent to which a PPP may support the public administration in carrying out its functions and in achieving public sector goals depends both on the institutional coherence of the adopted organisational form and the appropriateness of its governance and managerial structure.

To analyse the mission of institutional PPPs and their governance and managerial features we carried out a retrospective and evaluative exam of all of the cases of institutional PPPs established in the Veneto Region, one of the most developed areas of Italy in terms both of socio-economic indicators and quality of public services. We chose to study cases in the same region, to hold the characteristics of institutional context (health-care policy and legal framework) constant, thus highlighting more clearly the features of the single cases.

### Theoretical Framework

PPPs have come to the attention of scholars in a variety of research fields as potential instruments for channelling collaboration in traditionally public sectors. A number of scholars in the public management realm have highlighted the theoretical foundations of PPPs[3]. A first stream of research has conceived PPPs as a tool for fostering the privatisation of government functions through the delegation of public-service provisions, especially those that are infrastructure-based [2,4-6]. Conversely, sociological approaches to partnership [7,8] have underlined the importance of PPPs as a new public-governance paradigm to improve the efficiency and effectiveness of public service delivery. As such, their functioning requires not only a well-structured contract mechanism aimed at reducing transaction costs - as contended by new institutional economics perspectives [9-11], but also a degree of mutual trust between the parties [12].

Despite a surge of interest in the intellectual roots of PPPs, empirical literature has largely concentrated on the evaluation of contractual partnerships, with specific reference to private finance initiatives (PFI), either for country-specific experience [13-17] or in the context of comparative accounts [18]. In this perspective, analyses tend to assess a partnership's effectiveness primarily in terms of financial sustainability on the basis of value-for-money estimates.

However, institutional PPPs - formal legal entities run by proper corporate governance mechanisms and jointly owned by public and private parties for the provision of public goods - have received relatively little attention from public management researchers. In particular, the question of the extent to which these forms of stable partnership can ensure the sustainable provision of public services is still under investigated.

Drawing from these premises, the article aims to examine institutional PPPs with reference to two research questions: *What are the reasons for the adoption of institutional public-private partnerships? What governance and managerial features are necessary to make them appropriate arrangements for public service provisions?*

To address these research questions, a multiple embedded case study analysis of a population of institutional partnerships that emerged in the Italian health care system (*Sistema Sanitario Nazionale, SSN*) is presented. By doing so, this analysis contributes to the construction of a middle-range theory of institutional PPPs in public tax-funded health systems. Sections 2 and 3 describe the empirical setting and methods, respectively, and Section 4 presents the results. Section 5 provides a discussion of the findings, and Section 6 draws conclusions from the analysis.

### Empirical Setting

The research focused on the Italian National Health Service (*SSN, Sistema Sanitario Nazionale*) in which lawmakers in 1992 provided for the possibility of introducing institutional partnerships between public and private parties (DLgs 502/1992) to perform direct health-care duties - the so-called Joint-Provision-Form Experiments *(Sperimentazioni Gestionali,-JPFE*). JPFE can assume different legal configurations, including stock companies, limited companies, foundations, and associations. The unifying factor of this organisational population is that public and private constituents are involved in the production of a complex public good, health care service, albeit with different foci of activity, e.g., acute or rehabilitative hospital services, outpatient care, laboratory services. In this way, JPFEs differ from the other types of public-private partnerships that have emerged in the public sector, where either the service is contracted-out to the private sector, or the involvement of the private sector is limited to the construction of the facilities and the management of ancillary services (e.g. private finance initiative).

Public parties must retain majority shareholding (51%), and specific mechanisms should be introduced to limit the transfer of private shares to a third party. JPFEs are originally set up for a defined 3 (+3)-year period, at the end of which an external evaluation committee deliberates on either the liquidation or the confirmation of the settlement. With the exception of a minor update regarding those JPFEs established as stock companies (Lgs.D.229/99), the national government has not provided any additional legislation on the matter. Further, in the 2001 Constitutional Reform, Italian regions were given jurisdiction over the authorization, adoption and monitoring of JPFEs within their jurisdiction. With the exception of Lombardy and Emilia-Romagna, however, most regions passively acknowledged the 1992 national legislation, thus leading to a legislative and normative vacuum on the issue. The consequences of this lack of political guidance on the evolution of PPPs is evident when looking at the heterogeneity of individual responses to the questions raised by this study, as reported in the Results section.

## Methods

### Research Approach

The research questions were addressed through a contextual and comparative embedded case study design [19]. To ensure the external validity of the findings [20], the research assumed the Italian region as its context, which shall be studied as an independent health care organisational field due to recent health reforms that introduced decentralization principles in the Italian public sector field. Saturation was achieved by including, as the unit of analysis, the entire population of JPFE organisations established in the regional context. Specifically, the region under study is Veneto (ca 4.9 mil inhabitants and an annual public health expenditure of 8.5 billion €) where the JPFE population currently comprises four cases: (i) an intensive-rehabilitation hospital (Case A), (ii) an orthopaedic centre (Case B), (iii) a primary-care and outpatient facility (Case C), and (iv) a community-care facility (Case D). Three types of partner were usually involved in the establishment of a JPFE, namely (i) local health authorities (LHA), which are large public health care organisations responsible for the health of an entire population in a given area (average size of 230,000 inhabitants and an annual budget of 390 mil € [21]); (ii) private health care providers; and (iii) local municipalities, which are representatives of the resident population.

The study is partially grounded in the action research tradition. One of the authors was member of the regional evaluation committee in charge of evaluating the four JPFEs in force within regional boundaries. This privileged position enabled close interaction with regional policy-makers and JPFE top managers in the research process, the use of experiential knowledge as critical asset in understanding the cases and a close link between the practice of the research and its impact on social reality [22].

### Data collection

We relied on two primary data sources, archives and interviews. We began by gathering extensive documentary information from both internal and external sources. The internal sources included the statutes of the companies, annual reports, financial and economic prospectuses including budgets and balance sheets, and board minutes. The external sources included regional council resolutions, services agreements with the local health authorities, and public tenders for the selection of the private party. Approximately 23 documents were located per JPFE, for a total of 95 documents and 1,706 pages. Semi-structured interviews were conducted with managing directors of the experiments and with board members. These interviews were complemented by on-site visits to the facilities. Internal validity is guaranteed by the triangulation of sources in the data-collection phase.

### Data analysis

Data analysis began with an in-depth analysis of each case through the lenses of the research questions [23]. We first compiled and read the cases independently and then performed a cross-case analysis, in which the insights from each case were compared with those from other cases to identify consistent patterns and themes [24]. Drawing from the literature, institutional partnerships were analysed on four dimensions and ten sub-dimensions on the basis of the focus (internal or external to the organisation) and the level (strategic or operational) of the analysis (Table 1).

**Table 1:** Dimensions of Analysis

	**Focus**	
	*External*	*Internal*
	
*Strategic*	(i) **Institutional Aim** • Reason for the creation of the institutional partnership • Types of activities performed	(ii) **Governance Structure** • Organisation legal entity • Type of private and public sector partner and relative ownership share • Level of capital resources conferred to the PPP • Board governance structure
**Level**		
*Operational*	(iv) **Relationship with the Purchasing Authority** • Type of relation with the Local Health Unit or Municipality	(iii) **Bureaucratic and Administrative Procedures** • Procedures for the selection of the private partner • Type of legislation adopted for procurement • Type of contractual arrangement with the workforce

## Results

Results from the comparative analysis are summarised in the current section. Each sub-section discusses a dimension of analysis.

### Institutional Aim

A major theme in the pre-establishment phase concern the motives of both public and private actors in creating the JPFE. In most cases, the establishment of the collaboration followed a public health plan/program reorganisation of the health delivery structure, usually through a rationalisation of the hospital network and reduction of extra bed-capacity in the local area. *Case A*, for example, used to be an acute care facility that was forced to close in the 90s when the regional plan gave a nearby hospital exclusive rights to provide general health services. To guarantee continuity of care, the decision was made to convert the old acute care hospital into a high-specialisation rehabilitative hospital. Similarly, the establishment of *Case B *was motivated by the need to preserve the health care activity of the otherwise redundant hospital existing in the local area. *Case C *was based on the reconversion of the old acute hospital into a social-welfare facility. In all cases, the involvement of the private partner was motivated by the public sphere as a way to access additional financial resources and generate know-how and economies of experience. Concurrently, from a private party's perspective, the new activity would enable optimisation of the company's cost structure (Case C), or expand the existing business to either new geographical locations or activities (*Cases A *and *B*).

A common underlying rationale for the choice of a joint organisational form, as opposed to a traditional contracting-out agreement concerns the complexity of the services delivered. Contractual arrangements tend to be adopted for services characterised by a greater ability to be standardised and greater output measurability, e.g., laboratory, diagnostic or ancillary services. In contrast, in the case of a highly complex mix of activities (e.g., hospitals, rehabilitation centres) difficulties may arise in setting up suitable instruments to control qualitative and quantitative service standards. Furthermore, questions arise as to whether an effective risk transfer to the private contracting party can indeed occur, because the public ultimately keeps the responsibility for (in) efficiencies of the private management and the accountability for the production of the service to the population.

Indeed, results show that the JPFE organisational form is chosen whenever partners aim to work jointly for the delivery of a complex mix of health services. For example, *Case A *is a rehabilitative hospital predominantly focusing on rehabilitative cardiology and medicine, an outpatient clinic and diagnostic radiology services. Conversely, *Case B *is a mono-specialist centre for rare diseases, including myelitis and tuberculosis of the bones. Furthermore, it represents the logistic platform for basic specialised outpatient services and hosts the local emergency department. Similarly, *Case C *delivers two major types of health service, serving as outpatient clinics for major specialties, including allergology, angiology, cardiology, surgery, dermatology, echography, rehabilitative medicine, orthopaedics and pneumology, and hosting the local therapeutic community for mentally disabled patients of the Department for Mental Health. Finally, the focus on the welfare activities also characterises *Case D*, whose main activities relate to daily residential services to the elderly and disabled. Specifically, the facility hosts an average of 40 day hospitals and 200 hospitalised patients, the majority of whom are not self-sufficient or are affected by degenerative diseases, such as Alzheimer disease.

### Governance Structure

Although national legislation does not restrict the specific type of legal entity, all JPFEs within the region were set up as stock companies with public-sector majority. In *Case A*, ownership evolved from a substantial balance (52% LHA vs 48% private) to a public-sector dominance (75% LHA, 1.81% municipality and 23.19% private). *Case B *was originally set up as a limited company but was then transformed into a stock company, with 51% of shares held by the LHA and 49% by the private party. Similarly, in *Case C *the LHA holds 51% of the shares, the local municipality an additional 1%, and the private partner the remaining 48%. Contrary to hitherto discussed experiences, *Case D *is the only example of a JPFE with total public-sector ownership. In this case, 67% of the shares are held by the local municipality and 33% by the Local Health Authority. The reason for the establishment of this partnership was to jointly manage health and social services for a specific subset of the population (the elderly) that would otherwise require the overlapping of competences of the municipality (welfare) and the local health unit (health).

The analysis of the legal and ownership structure is strictly related to that of equity and allocation of capital resources to the newly established company. Of the four experiences, only *Case A *has been allocated considerable capital resources by the partners, enabling greater levels of financial autonomy and fostering infrastructural development. Indeed, the Local Health Authority conferred the building where the previous public hospital was located (6.2 mil €), whereas the private party conferred financial liquidity for c.ca 2 million €. In contrast, in the other JPFEs, the public-sector partner retained the ownership of the buildings, and the society pays a rent for the use of the facilities. For example, in *Case B *the society pays to the local LHA an annual rent of 2.5% of the value of the facilities. In *Case C *the facilities are given in commodatum and the society pays a token of 100 € to the LHA, whereas in *Case D the *facilities are owned by the local municipality.

As stock companies, all JPFEs are governed by boards composed of representatives of both parties, with duties of long-term strategic planning and budget approval. In *Case A *the board is composed of 9 members, 5 nominated by the public-sector parties and 4 by the private parties, while the managing director is nominated by the LHA out of a tern expressed by the private party. Similarly, in *Case C *the board is composed of 5 members, 3 of which (including the President) are an expression of the public sphere, while the Managing Director is nominated by the private partner. This scheme resembles *Case B*'s governance structure. Finally, in *Case D *the Board is composed of 3 members, 2 appointed by the local municipality and 1 by the Local Health Authority.

### Bureaucratic and Administrative Procedures

The selection process of the private partner is common to *Case A, B *and *C*. The initiative for the establishment of the JPFE originated within the public sector realm and the private partner was selected through public tender with a negotiated procedure. Criteria for admission included relevant previous experience in the health sector, volume of activity and workforce. Offers were assessed on the basis of price and quality criteria of the partnership project and on the potential accomplishment of specific public-sector goals, such as citizen orientation in health services (*Case C*). It might be discussed whether proper competition has in any instance really taken place in the selection of the private partner. Indeed, the analysis showed an average of two or three offers per tender, and the awarded offers were usually selected because of a lack of compliance of all others with one or more admission criteria. As will be discussed in the next section, this detail might influence the generalisability and transferability of the JPFE model to other settings.

The lack of specific regulation on the type of legislation, administrative or civil, JPFEs should follow in the performance of daily activities led to a heterogeneity of solutions adopted to carry out the procurement function. Indeed, *Cases C *and *D *follow the administrative law of public sector organisations, implying that purchasing activities are carried out by public tender procedures aimed at guaranteeing transparency and impartiality of the process. In contrast, *Cases A *and *B *follow the civil law of private companies to ensure higher flexibility and speed in the purchasing process. The former experience has, however, developed and implemented a voluntary Code for Purchases that has been recently proposed as the standard at the regional level for future JPFEs.

Heterogeneity has also been found in the treatment of the workforce. In *Cases C *and *D *the workforce is entirely employed by the new public-private company. In the former case, employees of the former public hospital were transferred to other public facilities within the same Local Health Unit. Administrative staff are employed by the company, doctors hold private-practice contracts, whereas nurses and other non-professional are employed by a contracted-out cooperative. On the contrary, in *Cases A *and *B *the personnel previously operating in the former public hospital were given the option either to work in the new public-private facility or be transferred to another public hospital. Although a discrete majority opted for the latter option, part of the professional workforce decided to remain, thus leading to the emergence of a heterogeneity of professional figures within the organisational boundaries. Indeed, public professionals with a permanent employment contract maintain the employment relationship with the LHA and the SSN contract. In contrast, professionals employed after the establishment of the partnership hold the national contract for private practice (AIOP). Should the outcome of the 3(+3)-year partnership prove positive, public professionals will then be employed by the company. Otherwise, they will be reintegrated in the LHA.

### Relationship with the Purchasing Authority

The relationship with the purchasing authority plays an essential role in determining the nature of the activities performed by the JPFEs. In all cases, a service contract was agreed upon the company and the public purchaser, typically the Local Health Authority or the municipality. The length of the agreement spans from a fixed temporal period (*Case A*) to the entire duration of the experiment (*Case B*). In *Case B*'s agreement, the local health authority entrusted the integrated system of health-welfare services to the company, explicitly prohibiting the subcontracting to third parties. In *Case A *distinct sub-sets of conventions were signed (e.g., for the blood centre, and cardiology rehabilitation), and the total volume of agreed activities agreed sharply increased, moving from 7 million € in 2004 to 21 million € in 2008. Finally, in *Case C *outpatient services are fully integrated with those of the Local Health Authority: the planning of the volume of activities of the JPFE is indeed a sub-set of the budget of the LHA.

## Discussion

This section discusses the reported findings and provides the basis for answering the research questions, namely the reason for the adoption of institutional PPPs as complementary provision forms, and the governance and management features they need to be effective. We follow the sequence of Table 1.

### Institutional Aim

Two possibly conflicting goals led to the establishment of all the JPFEs. The first is a strategic goal, namely, the rationalization of the public hospital network and the subsequent dismissal of a small general hospital. The second, however, is the policy goal of maintaining the local political consensus through the conversion of an existing hospital into a different type of healthcare facility that can still be regarded as part of the public realm and that serves the local population. A major issue in the establishment phase was therefore the transformation of the health care generalist orientation and the definition of a portfolio of highly specialised services. To implement the new policy and develop the entrepreneurial model, the public sector opened up to collaboration with private parties. Indeed, in all cases, private partners were involved not only in financing the investments for infrastructural renovation, but also in carrying out niche activities, because they are supposed to hold stronger managerial competences.

Two consequences flow from the above arguments. First, the underlying reason for the creation of institutional PPPs was the need to find a politically viable solution to the introduction of cost-cutting health care policies (and the consequences deriving from their implementation, e.g., the dismissal of small local general hospitals), rather than the result of rational planning to enhance forms of public-service provisions. Second, institutional PPPs were not conceived as alternatives, but rather as complement to the traditional public-service provisions for a defined set of services and goals. Indeed, two empirical considerations support these arguments. First, each JPFE budget is part of the general LHA budget. The public authority thus monitors JPFE expenditure and ultimately guarantees any financial risks taken over by the company. Second, JPFE's missions were designed to lessen the pressure on the LHA budgets through the development of profitable market segments that would increase the demand for both SSN inbound patient mobility and private out-of-pocket patients.

All cases achieved the real (hidden) institutional aim, namely the dismissal of a small hospital without major complaints from the local public.

### Governance Structure

All JPFEs were set up as stock companies, suggesting the will of public partners to operate in a more flexible legal framework than that of the traditional public-sector regulation. In all cases, private partners are entrepreneurs with different degrees of specialisation and organisational development in distinct branches of healthcare. What is intriguing is the extent to which JPFEs were granted autonomy by the constituent partners. Indeed, public parties held two contradictory expectations, namely the development of a dynamic and entrepreneurial initiative and the need to maintain the direct control over the service delivery. Concurrently, private partners wanted to retain responsibility for the entrepreneurial and managerial functions despite their minority shareholding.

In this potentially conflicting scenario, the degree of capitalisation of the JPFEs played a pivotal role in determining the effective degree of autonomy of the PPPs. Only in *Case A *were the assets transferred to the new society by the public party as a form of capitalisation, while the private partner contributed significant financial resources to balance the level of investment and, consequently, of shares. In doing so, the organisation was granted autonomy to develop infrastructural activities, one of the major drivers of strategic change in the health care sector. Both infrastructural transformation and services' portfolio reorganisation plans were enabled by the high degree of capitalisation. The relevant financial exposition of both investors constituted a strong incentive for the JPFE management, which was asked to guarantee an adequate rate of return on the investments. However, the return on investment for the private partner was squeezed by the public shareholding, even though the latter originated exclusively from the allocation of physical resources. This structure made the financial constraints brought in by the private investor more difficult.

In contrast, in the remaining cases, LHAs owned the facilities and rented them to the PPPs, thus partially undermining JPFEs' strategic autonomy and weakening management commitment to results. Furthermore, this strategy brought the public partner back in the organisational internal decision process due to mandatory approval needed to perform any investment activity.

Generally speaking, a chief reason for the lack of allocation of public capital resources (e.g., buildings and facilities) to the PPP is that the amount of capital transferred depends upon the value of the single assets, which may not reflect the amount needed for the agreed division of shares. While private partners may achieve balance in equity shares only by increasing the financial contribution, this action can lead to an overcapitalisation of the company compared with the estimated turnover in service activities. However, despite these limitations, results have shown that whenever physical assets were transferred to the newly established company, they acted as a strong organisational incentive to achieve PPPs' strategic goals by partially increasing managerial autonomy.

### Bureaucratic and Administrative Procedures

Public tender procedures have been adopted in all cases for the selection of the private party. The stringent criteria set for the evaluation of offers, however, limited the number of firms participating in the selection process, and often led to the submission of a single offer. The lack of private-sector initiative unveiled the difficulty in finding private entrepreneurs willing to cooperate with PAs in service-delivery functions (and not only in financial or building activities). This issue is common to many institutional PPPs throughout Europe and is generally due to both market factors (lack of private-sector entrepreneurs focusing on core health services) and procedure limitations (difficulty in establishing a negotiation based on trust).

From an administrative point of view, despite the common legal framework of the stock company, two JPFEs continued to use public procurement procedures in the fulfilment of ordinary purchasing activities, while the other two adopted private procedures. The driver behind this latter choice can be traced back to the internal organisational culture and, specifically, to the degree of autonomy given to the managers appointed by the private partner. Only in *Case A *has a proper public-private integrated purchasing approach been developed, through the adoption of a voluntary code regulating private buying procedures and guaranteeing transparency and publicity in the procurement process. This arrangement constitutes a good example of the virtuous coexistence of the two cultures.

A final issue concerns the relationship with the workforce, with specific regard to the contractual relations. Civil servants operating in the previous facilities and continuing their work in the new company kept the public-sector contracts even though they would have been required by law to shift to the private discipline. The co-existence of personnel with different contractual profiles for the same job positions, although justified by the novelty of the experience and the need to maintain political consensus, may lead in the long term to treatment inequalities and internal resistance from part of the workforce. This findings shows the "provisional" nature given to the JPFE and the difficulty in formalizing the collaboration through a stabilised institutional form.

### Relationship with the Purchasing Authority

In all cases, the public partner (i.e., LHA) was both the owner of the company (51% shareholding) and the purchasing authority (almost 100% of revenues in all cases). This indeed reflects two possible ways in which the public sector can exert control over the PPP, namely through ownership or purchasing powers. Acting as the purchaser is potentially easier when the hospital has a clear and focused mission and provides a limited set of services, as in the case of the JPFEs, where initial service agreements were signed, specifying service targets and revenues. Conversely, whenever the mission is broad and largely indefinite, as in the case of general hospitals, it is rather difficult to develop the purchasing function. However, in all cases analyzed, the public authority preferred to act through the property function, steering and latently influencing the decision-making processes at the PPP board level rather than through the more politically visible inter-institutional purchaser function.

### Final Reflections on the Initial Performance

*Case A *has registered an annual 18% increase in the number of discharges in the 2004-2008 period thanks to the implementation of a strong network with the cardiology and cardio-surgery acute departments within the area. The high levels of achieved specialisation achieved led to an increase in the mix of patients coming outside the sphere of competence of the Local Health Authority (currently 50% of the users). The significant expansion of activities and revenues was agreed by the board, where the public partner acted as shareholder, without previously modifying the service contract agreed to. Thus, the LHA purchasing function was not properly activated, because the adjustments on the service agreement were rather made as an ex post bureaucratic duty. Conversely, *Case B *has only partially achieved the activity targets, and failed the pre-established targets of infrastructural renovation. Financial indicators show a precarious economic situation, with substantial losses in the 2007-2008 biennium (-471,000 €). Despite the intense debate raised at the board level, no action was taken by the purchasing authority, which did not adjust the contract goals and revenues for the subsequent years.

In a similar way, the remaining two JPFEs report a breakeven over the whole period. However, they failed to develop the new expected service areas. The LHAs in their purchaser role were not able to exert pressure on the organisation, being rather satisfied as shareholders to break even. The weakness of the purchasing function may also explain the lack of infrastructural investments undertaken by the public partners owning the facilities, being a signal of an unclear target planning.

## Conclusions

The overarching aim of the paper was to investigate the reasons for the adoption of institutional PPPs and the governance and managerial features that could make them effective. The evidence collected from the four cases revealed that public administrations did not follow a rational decision process in the type of provision form deemed most efficient for a given set of services. Rather, PPPs were chosen as contingent solutions to implement health policy reforms without losing local political consensus. Stated differently, PPP were conceived as a way of renovating public facilities through the involvement of private providers without losing public sector control. Specifically, the choice of this organizational form was adopted to enable the conversion of hospitals for general care, whose missions and goals are broad and hardly definable, into specialised care facilities. In all four cases, the (hidden) policy goal of partially dismissing a local hospital while maintaining stakeholder consensus was achieved.

All PPPs were established with clear and focused goals so that the relationship between the public-sector authority and the company could be regulated by service agreements specifying the activity targets, the correspondent fees and revenues, and, consequently, the return on the investment. This arrangement could have made the purchasing function a potential key steering tool. However, in all cases, the ownership function was the first to be activated, and public partners preferred to act as company owners rather than service commissioners, even if targets were measurable and defined. The former was exerted in the board rooms and was therefore less politically visible, while the latter was rather public and transparent.

In general terms, those PPPs that obtained a positive evaluation by the regional committee shared four governance and managerial features: (i) a strategic market orientation to a specialised service area with a sufficient potential demand, (ii) the allocation of public capital assets and the consistent financial involvement of the private partner, (iii) the adoption of private administrative procedures in a regulated setting while guaranteeing the respect of public administration principles, and (iv) a clear regulation of the workforce to align the contracts with the organizational culture. In contrast, the lack of significant capital involvement and, consequently, of partners' commitment, combined with the maintenance of public administrative procedures and the presence of unclear contractual arrangements may explain the unsatisfying results of some of the experiences.

To conclude, the institutionalization of the collaboration between public and private actors by the establishment of a new independent company highlights two major tradeoffs deriving from bringing different public and private agendas into an organised setting. The first is private for-profit seeking versus public breakeven maintenance. Our research suggests that this trade-off might be overcome by considering returns in terms of intangible profits such as image and economies of experience deriving from the privileged position the private partner holds within the SSN. The second is public local budget containment versus private portfolio and scale increase. Recent trends for JPFEs suggest that this conflict can be resolved by rethinking about public shareholding, namely by diversifying the mix of public partners including multiple local authorities so that the PPP can increase the level and amount of commissioning.

## Competing interests

The authors declare that they have no competing interests.

## Authors' contributions

GC and FL jointly developed the design of the study. They also interpreted and analysed the data. All authors read and approved the final manuscript.

## Pre-publication history

The pre-publication history for this paper can be accessed here:

http://www.biomedcentral.com/1472-6963/11/82/prepub
